# Enhancing Screening and Counseling for Alcohol Use Disorder in a Predominantly Hispanic Border Town Population With the Alcohol Use Disorders Identification Test-Concise (AUDIT-C)

**DOI:** 10.7759/cureus.84541

**Published:** 2025-05-21

**Authors:** Michael J Brockman, Bhavi Trivedi, Lakshmi Kattamuri, Fabrizzio Delgado, Lisa A Hechanova, Abhizith Deoker, Brian P Edwards

**Affiliations:** 1 Internal Medicine, Texas Tech University Health Sciences Center El Paso, Paul L. Foster School of Medicine, El Paso, USA; 2 Internal Medicine, Texas Tech University Health Sciences Center El Paso, El Paso, USA; 3 Psychiatry, University of Texas Southwestern Medical Center, Dallas, USA; 4 Internal Medicine/Nephrology, Texas Tech University Health Sciences Center El Paso, El Paso, USA

**Keywords:** alcohol misuse, alcohol use disorders (aud), general internal medicine, preventative care, screening test

## Abstract

The United States Preventive Services Task Force (USPSTF) recommends routine screening for alcohol use disorders in adults 18 years or older using one to three validated tools. One favored tool is the Alcohol Use Disorders Identification Test-Concise (AUDIT-C), a brief three-item questionnaire noted for its ease of implementation, sensitivity, and specificity. A positive screen on the AUDIT-C should prompt brief behavioral health counseling to help reduce harmful drinking patterns.

Before this quality improvement project, the Texas Tech University Health Sciences Center (TTUHSC) El Paso Internal Medicine Residency Clinic did not routinely use the AUDIT-C and its residents had not received training in alcohol-related behavioral health counseling. In response, the AUDIT-C was integrated into the clinic’s electronic medical record (EMR), and residents received instruction on alcohol use disorder counseling from a certified physician in Addiction Medicine. This intervention enhanced compliance with USPSTF recommendations and provided an opportunity to evaluate alcohol use in a primarily Hispanic border population.

A total of 39 patients reporting alcohol use were screened with the AUDIT-C; 34 screened positive and five screened negative. Among the patients who received behavioral health counseling, 12 completed a post-counseling survey to assess their drinking habits. The survey revealed that 10 (83%) of these patients had never previously received counseling. Furthermore, nine (75%) found the counseling helpful, eight (67%) were surprised by the unhealthiness of their drinking habits, and nine (75%) felt comfortable during the session. Additionally, eight (67%) expressed a willingness to consider changing their drinking behaviors. Among those unwilling to change, five patients did not perceive a problem with their drinking, while one patient did not wish to reduce alcohol consumption.

These findings demonstrate a strong willingness among El Paso residents to discuss problematic alcohol use within the clinical setting. The positive patient responses support the broader implementation of the AUDIT-C in practice and underscore the importance of training clinicians in effective intervention techniques.

## Introduction

Alcohol use disorder (AUD) is defined by impaired control over alcohol consumption, leading to negative health effects and difficulty performing daily activities. While alcohol consumption itself carries inherent health risks, organizations have established specific criteria to classify AUD. The National Institute on Alcohol Abuse and Alcoholism (NIAAA) recommends that males under 65 consume no more than 14 standard drinks per week and no more than four drinks per day, while females and males aged 65 and older should not exceed seven drinks per week or three drinks per day [1]. The Diagnostic and Statistical Manual of Mental Disorders, Fifth Edition (DSM-5), defines AUD as a problematic pattern of alcohol use causing significant distress or impairment within a 12-month period [2].

Globally, AUD remains a major public health concern, with the World Health Organization (WHO) estimating that alcohol-related deaths exceed 3 million annually. Among individuals aged 15-49, alcohol-related disorders are the leading cause of premature death and disability, accounting for 10% of all fatalities in this age group [3]. In the United States, the Centers for Disease Control and Prevention (CDC) ranks Texas among the states with the highest binge drinking rates and the second-most economically impacted state due to alcohol consumption [4,5].

In response, the U.S. Preventive Services Task Force (USPSTF) recommends screening all adults aged 18 and older, including pregnant women, for AUD in primary care settings, along with behavioral counseling interventions to reduce alcohol use [6]. One commonly used screening tool, the Alcohol Use Disorders Identification Test-Concise (AUDIT-C), helps detect problematic drinking, and individuals who screen positive are encouraged to seek behavioral interventions such as counseling [7]. However, multiple barriers, including stigma, privacy concerns, time constraints, and cultural or linguistic differences, can hinder effective screening and intervention.

El Paso, Texas, a multicultural, trans-border city adjacent to Juárez, Mexico, presents a unique setting to evaluate the effectiveness of USPSTF recommendations. As one of the busiest border crossings in the world, El Paso’s international dynamic poses challenges for implementing AUD screening, and there is limited knowledge regarding how patients in a predominantly Hispanic border community perceive AUDIT-C screening and its impact on identifying and addressing AUD [7,8].

This study hypothesizes that incorporating the AUDIT-C questionnaire and training residents in effective AUD counseling will increase awareness of alcohol use disorder among patients. Implementation at Texas Tech University Health Sciences Center (TTUHSC) El Paso Internal Medicine Clinic is expected to enhance adherence to USPSTF screening recommendations and improve patient outcomes.

The primary objective of this study is to increase AUD awareness and establish early interventions within the clinic in accordance with USPSTF guidelines. Additional anticipated benefits include increasing the rate of AUD screening, education, and counseling, improving awareness of AUD among patients and providers, assessing barriers to seeking AUD-related interventions, and identifying the prevalence of AUD within the clinic population.

## Materials and methods

This quality improvement project was designed as a cross-sectional study conducted at the TTUHSC El Paso Internal Medicine Clinic. Institutional Review Board (IRB) approval was obtained in January 2023, and the study was conducted in accordance with the Declaration of Helsinki. The IRB approved the integration of the AUDIT-C questionnaire into the clinic’s electronic medical record (EMR) system, along with the implementation of a post-counseling survey to assess patient perspectives on alcohol use disorder counseling. Both the AUDIT-C questionnaire and the post-counseling survey were provided in English and Spanish to accommodate patient language preferences.

Prior to study initiation, a target sample size of 100 patients was determined based on a power analysis aimed at detecting a medium effect size (Cohen’s d=0.5) with an alpha level of 0.05 and a power of 80%. This analysis suggested that a minimum of 64 participants per group would be required for statistically robust comparisons. However, the final sample size was limited by the number of patients who visited the clinic and consented to participate, resulting in a lower-than-target enrollment.

The study was conducted over a three-month period, from January 9 to April 10, 2023. Inclusion criteria encompassed all patients aged 18 years or older who consumed alcohol and were screened using the AUDIT-C questionnaire. Patients who did not report alcohol consumption were excluded from screening and the study. Participants were allocated to the study group based on these predefined inclusion and exclusion criteria without randomization.

The AUDIT-C questionnaire in Appendix 5 served as the primary screening tool for identifying alcohol use disorder (AUD). It consisted of three questions assessing alcohol consumption patterns over the past year. The first question asked about the frequency of alcohol consumption, with response options ranging from Never to 4 or more times a week, each assigned a score from 0 to 4. The second question evaluated the number of standard drinks consumed on a typical drinking day, with responses from 1 or 2 drinks to 10 or more, also assigned point values from 0 to 4. The third question assessed the frequency of consuming six or more drinks on one occasion, with response categories from Never to Daily or Almost Daily, again scored from 0 to 4. The total AUDIT-C score was calculated by summing the individual responses. A score of less than 3 was classified as non-problematic alcohol consumption, while a score of 3 or greater in female patients or 4 or greater in male patients was considered a positive screen for alcohol use disorder, indicating the need for further evaluation or intervention.

Additionally, a Mann-Whitney U test was employed to compare the distribution of AUDIT-C scores between male and female patients who screened positive. This non-parametric test was selected because the score data did not conform to a normal distribution, thus providing a robust measure of difference between the groups.

Prior to the study, the TTUHSC El Paso Internal Medicine Clinic did not routinely implement USPSTF recommendations for alcohol use disorder screening. As part of this quality improvement initiative, the AUDIT-C questionnaire was integrated into the clinic’s EMR, replacing the previous screening method. Additionally, Internal Medicine residents underwent specialized training in alcohol use disorder counseling, conducted by a certified physician in Addiction Medicine. The interactive sessions focused on teaching residents appropriate counseling techniques, including motivational interviewing and brief intervention strategies tailored to patients with AUD. The training aimed to equip residents with the necessary skills to provide effective behavioral health interventions for patients who screened positive for AUD.

During routine clinic visits, patients were asked about alcohol consumption at the time of triage. Those who reported any alcohol use, regardless of frequency or quantity, completed the AUDIT-C questionnaire. If the screening result was positive, the patient was offered voluntary behavioral health counseling, provided by their Internal Medicine resident physician. Following the appointment, patients were given the option to complete a voluntary post-counseling survey (Appendix 6), available in both English and Spanish, to evaluate their attitude toward counseling, willingness to engage in further interventions, and perceived barriers to enrolling in alcohol use disorder treatment programs. The survey responses provided insight into patient perspectives on AUD screening and intervention, helping identify potential areas for improvement in implementing screening guidelines.

To evaluate the effectiveness of the intervention, the frequency of AUDIT-C questionnaire administration and follow-up behavioral health counseling sessions was recorded and analyzed. Data from the post-counseling survey were also collected, with results tabulated and visualized using pie charts and bar graphs to illustrate screening rates, counseling participation, and barriers to intervention. These analyses provided a comprehensive assessment of the study’s impact on adherence to USPSTF recommendations and its effectiveness in increasing awareness of alcohol use disorder within the clinic population.

## Results

**Figure 1:**
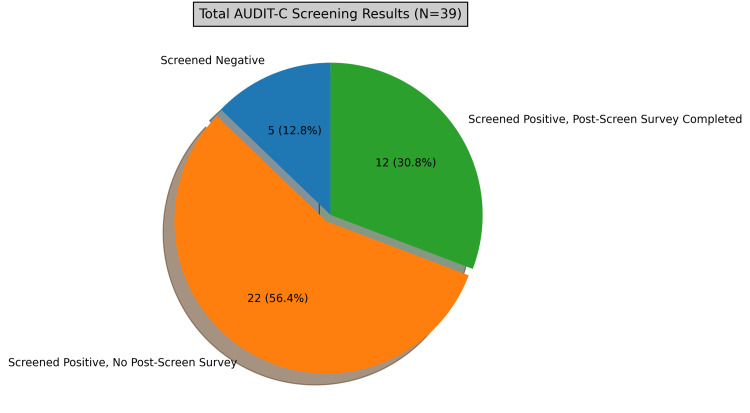
Representation of AUDIT-C responses. ‘Screened Negative’ represents patients who denied alcohol consumption. ‘Screened Positive, No Post-Screen Survey’ represents patients who reported alcohol consumption however did not complete the post-counseling survey. ‘Screened Positive, Post-Screen Survey Completed’ represents patients who reported alcohol consumption and completed the post-counseling survey.

**Figure 2:**
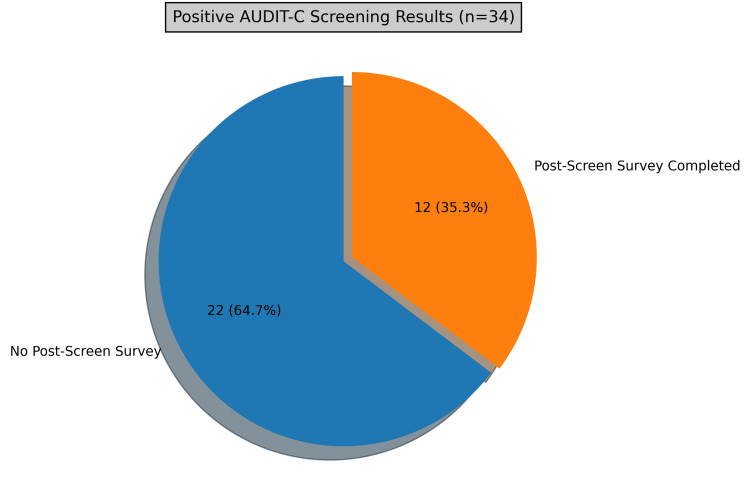
Representation of patients who completed and who did not complete the post-counseling survey.

During the three-month period, 39 patients (mean age 44 years) were administered the AUDIT-C survey following self-reported alcohol use. A total of 34 patients (87%) screened positive for alcohol use disorder based on the AUDIT-C scoring criteria (score of >2 in females, >3 in males), represented in Figure 1. A total of five patients (12.8%) screened negative for alcohol use disorder. Of the 34 patients who screened positive, only 12 (35.3%) completed the post-counseling questionnaire, and 22 (64.7%) did not complete the post-counseling questionnaire (represented in Figure 2).

Of the total population who screened positive (n=34), the average AUDIT-C score was 4.4 (Mdn=3.5). When subdividing between genders who screened positive, female patients were found to have a lower average score (M=3.6, Mdn=3.0) than males (M=5.8, Mdn=6.0). The highest reported score amongst female patients (score=9) was found to be lower than males (score=11).

A Mann-Whitney U test was performed to compare AUDIT-C scores between male and female patients who screened positive. The analysis yielded a U-statistic of 44.0 and a p-value of 0.897, indicating no statistically significant difference in AUDIT-C scores between genders. These results suggest that, within this sample, the distribution of alcohol consumption severity, as measured by AUDIT-C scores, was similar for both male and female patients.

**Figure 3:**
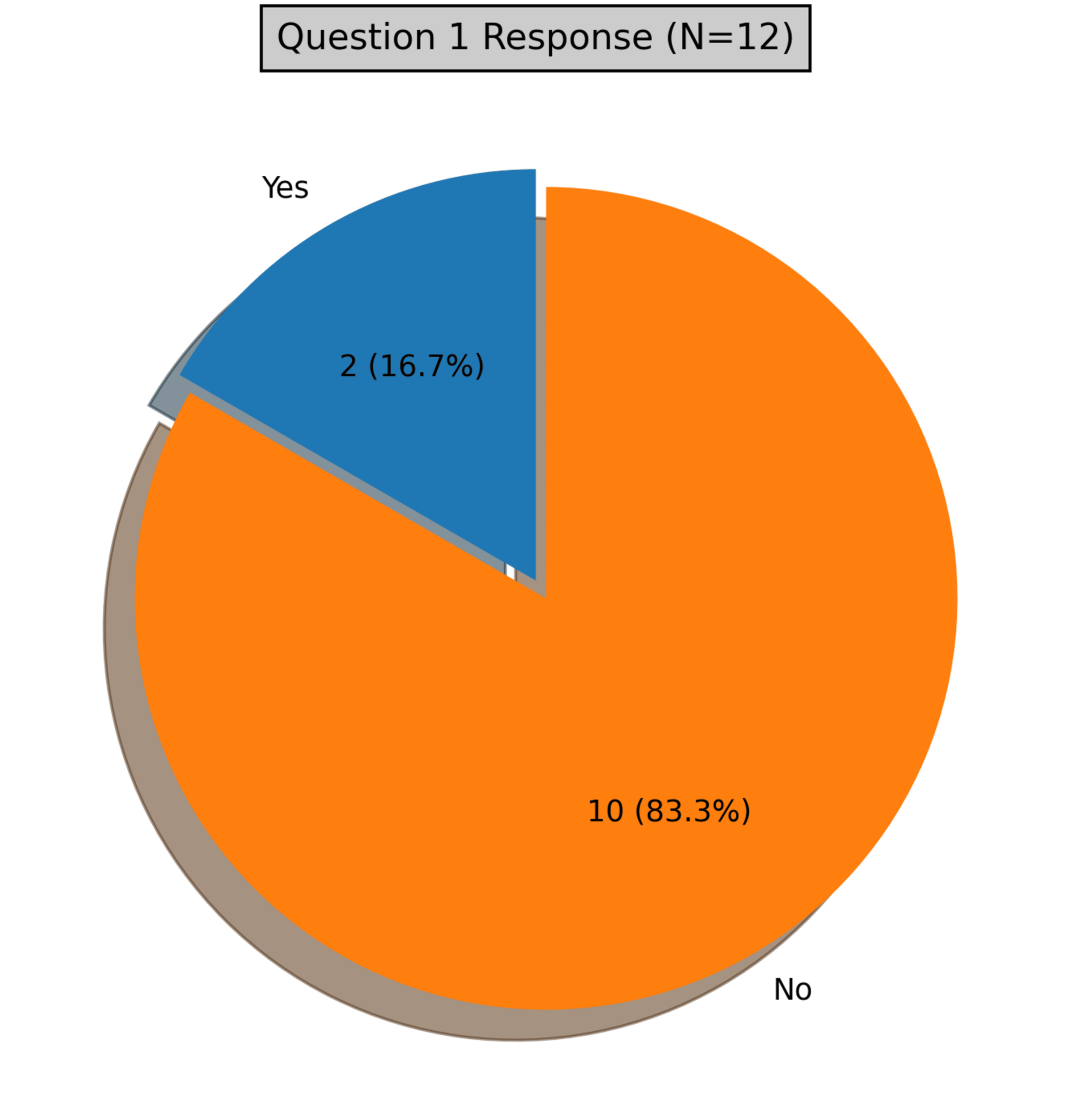
Graphical representation of question 1 of the post-counseling survey: Has anyone counseled you before regarding your drinking habits?

From the post-counseling survey, most patients reported having never received behavioral health counseling with regards to alcohol consumption (n=10; 83.3%), while two (16.7%) reported having received counseling in the past, as represented in Figure 3. The post-counseling survey also revealed that the majority of patients found the counseling helpful and educational (n=9; 75%). Many of the patients were surprised that their drinking habits were unhealthy (n=8; 67%).

**Figure 4:**
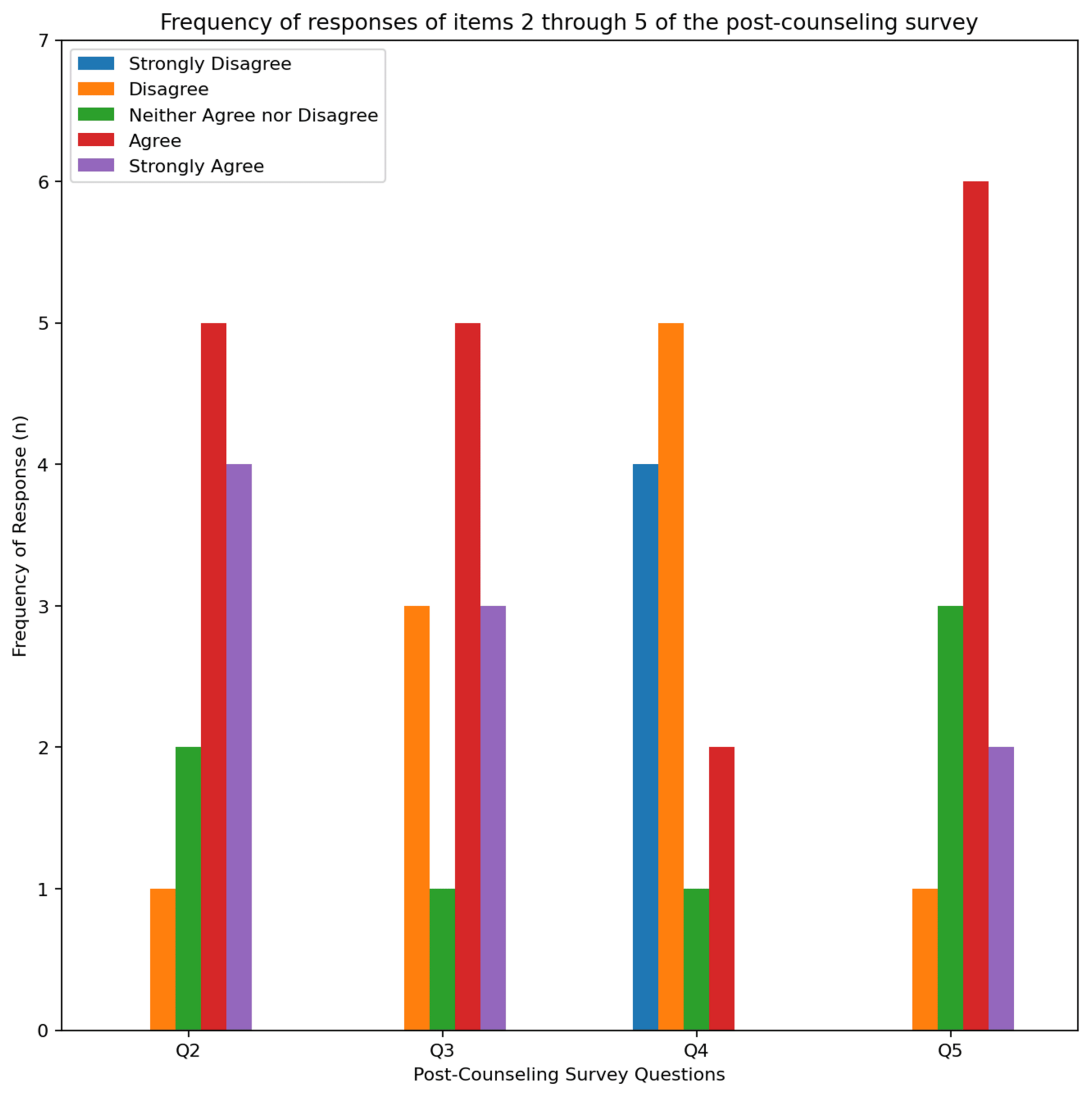
Frequency of responses of items 2 through 5 of the post-counseling survey. Items 2 through 5 are as listed: (2) this conversation provided education on alcohol drinking habits, (3) it was surprising to you that your alcohol drinking habits are considered unhealthy, (4) discussing your alcohol use habits made you uncomfortable, (5) will you be implementing changes to your drinking habits?

As for attitudes towards behavioral health counseling, most patients stated they did not feel uncomfortable during the counseling (n=9; 75%). For assessing patients’ attitudes towards changing their habits, (n=8; 67%) patients were willing to institute change. Of those who reported that they were unwilling to change, five reported they did not find an issue with their drinking, and one reported they did not want to cut down on drinking. The responses of items 2 through 5 of the post-counseling survey are graphically represented in Figure 4.

## Discussion

Alcohol-associated mortality is rising in the United States, necessitating difficult yet essential discussions with patients about mental and behavioral health [9,10]. Given the increasing morbidity and mortality linked to alcohol use, adherence to the U.S. Preventive Services Task Force (USPSTF) recommendations has never been more critical. This quality improvement project aimed to address these concerning trends by enhancing the TTUHSC El Paso Internal Medicine Clinic’s compliance with USPSTF guidelines for alcohol use disorder screening. Additionally, the study sought to evaluate patient attitudes toward behavioral health counseling for alcohol use and identify barriers that may have hindered patients from seeking help in the past.

The project successfully increased screening rates in the clinic, achieving a higher rate of alcohol use disorder screening compared to similar studies [11,12]. This is particularly relevant given that national estimates suggest that screening rates for alcohol use disorder remain suboptimal, reported to be as low as 46% in the United States [13]. The findings from this study demonstrate a feasible and effective strategy for improving alcohol use disorder screening in a primary care setting.

Similarly, the rate of behavioral health counseling in the clinic exceeded national estimates, which indicate that fewer than 25% of patients who screen positive for alcohol use disorder receive counseling [14]. One study found that only 39.7% of patients who screened positive were offered counseling, with patient resistance cited as a primary reason for low counseling rates [15]. A similar trend was observed in a study conducted by the United States Department of Veterans Affairs Health System, where approximately 30% of patients with high-risk alcohol use received psychosocial intervention [16]. The improved counseling rates observed in this project suggest that integrating behavioral health counseling into routine clinical care may enhance patient engagement and willingness to participate in interventions.

Concerns regarding how TTUHSC El Paso Internal Medicine Clinic patients would respond to behavioral health counseling were alleviated by overwhelmingly positive feedback. The post-counseling survey revealed that 75% of patients found the intervention helpful. Additionally, most patients reported never having been screened or counseled for alcohol use disorder prior to this study, highlighting a significant gap in patient care. These findings suggest that routine alcohol use disorder screening and counseling may be underutilized despite existing national guidelines recommending their implementation.

This quality improvement project offers valuable insights and serves as a template for improving alcohol use disorder screening protocols within a residency clinic setting. Furthermore, it provides evidence of patients’ willingness to engage in behavioral health counseling when offered in a structured and supportive environment. Notably, this project was conducted in a predominantly Hispanic patient population, a demographic that continues to grow in the United States. Understanding the effectiveness of alcohol use disorder screening and intervention strategies within diverse populations is essential for addressing disparities in healthcare access and ensuring that all patients receive appropriate and timely interventions.

Limitations

Primary limitation resides in the small sample size. The relatively smaller sample size may restrict the generalizability of findings to a broader population and limit the statistical power of the study. To ensure credibility and reliability of study outcomes, future follow-up studies should be considered with larger and more diverse sample sizes. The study’s scope might not encompass the evolving dynamics of participants' attitudes and behaviors over extended periods, thereby restricting the ability to draw conclusions about the impact of counseling on sustained behavior change. This study emphasizes the need for screening for alcohol use disorder among adults with brief screening tools like AUDIT-C and proactive behavioral health counseling interventions for those who screen positive.

Future direction

Future research should include follow-up surveys to see if changes in alcohol use behavior persist after screening and counseling. This would help gauge the long-term impact of the intervention. It would also be valuable to replicate this study in more diverse populations to better understand how cultural factors and social determinants of health (SDOH), like comorbidities, education, and income, influence alcohol use and response to counseling. Exploring these areas could highlight barriers to care and lead to more personalized, effective interventions.

## Conclusions

The presented quality improvement project successfully enhanced the TTUHSC El Paso Internal Medicine Residency Clinic’s adherence to USPSTF recommendations for alcohol use disorder screening while also capturing patient perspectives on their alcohol use. The implementation of the AUDIT-C questionnaire and behavioral health counseling led to significant improvements in screening rates and intervention efforts, surpassing national averages. Furthermore, the post-counseling survey revealed that patients responded positively to behavioral health counseling and identified key socio-economic barriers that may hinder access to care. These findings highlight the effectiveness of integrating standardized screening and counseling into routine clinical practice and underscore the need for continued efforts to address patient-reported barriers, ultimately improving outcomes for individuals at risk of alcohol use disorder.

## Figures and Tables

**Figure 5:**
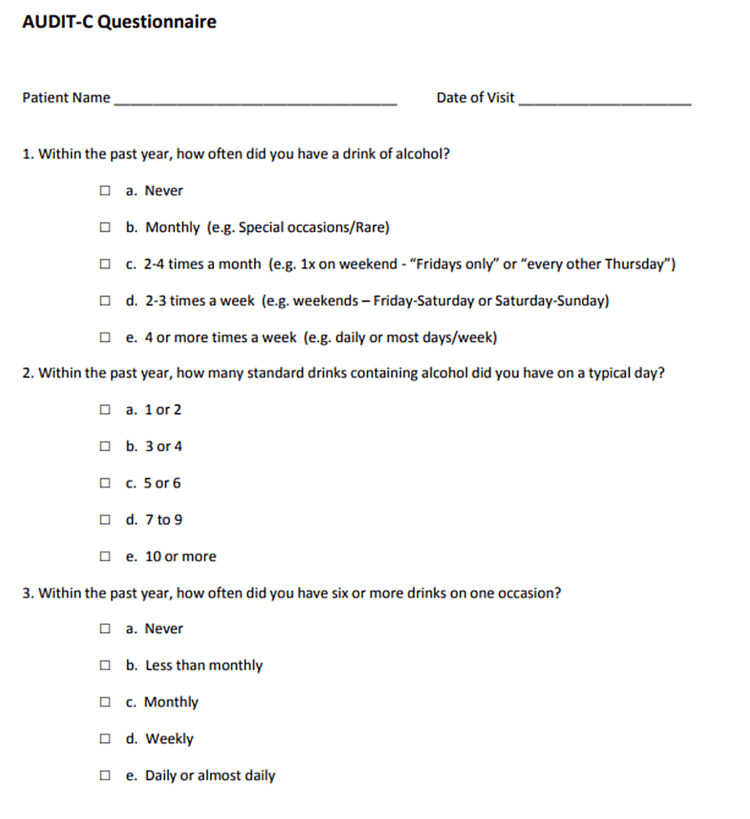
AUDIT-C survey (English). A digital implementation was integrated into the TTUHSC EMRs, using the same verbiage, structure, and scoring as presented. The AUDIT-C is a validated screening tool originally developed by the World Health Organization for the identification of hazardous and harmful alcohol consumption. This instrument is publicly available and was reproduced here based on its status in the public domain; hence, no additional permission was required for its use. For further details regarding the AUDIT-C, please refer to the World Health Organization’s website: https://www.who.int/substance_abuse/activities/audit/en/. TTUHSC: Texas Tech University Health Sciences Center; AUDIT-C: Alcohol Use Disorders Identification Test-Concise; EMR: electronic medical record

**Figure 6:**
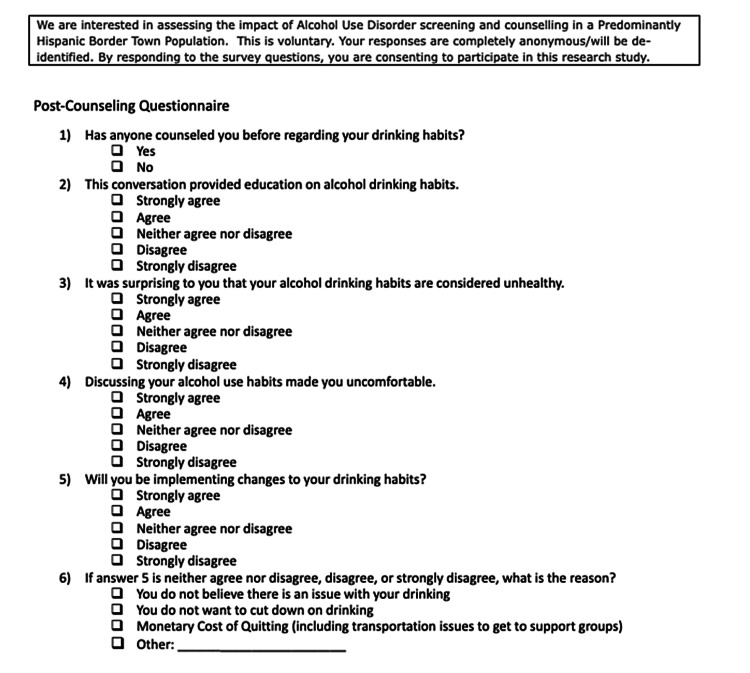
Post-counseling questionnaire provided to patients after behavioral health counseling. The questionnaire was provided in both English and Spanish.

## Appendices

Appendix 5

Figure 5 and Table 1 show the AUDIT-C survey.

**Table 1:** AUDIT-C, developed by the World Health Organization (WHO) The AUDIT-C is a validated screening tool originally developed by the World Health Organization for the identification of hazardous and harmful alcohol consumption. This instrument is publicly available and was reproduced here based on its status in the public domain; hence, no additional permission was required for its use. For further details regarding the AUDIT-C, please refer to the World Health Organization’s website: https://www.who.int/substance_abuse/activities/audit/en/. AUDIT-C: Alcohol Use Disorders Identification Test-Concise

Item	Question	Response Options	
Q1	How often do you have a drink containing alcohol?	0 = Never; 1 = Monthly or less; 2 = 2-4 times a month; 3 = 2-3 times a week; 4 = 4 or more times a week	
Q2	How many standard drinks containing alcohol do you have on a typical day when you are drinking?	0 = 1 or 21 = 3 or 42 = 5 or 63 = 7 to 94 = 10 or more	
Q3	How often do you have six or more drinks on one occasion?	0 = Never; 1 = Less than monthly; 2 = Monthly; 3 = Weekly; 4 = Daily or almost daily	

Appendix 6 

Figure 6 and Table 2 show the post-counseling questionnaire.

**Table 2:** Post-Counseling Questionnaire (English Version) Credits: Michael J. Brockman, DO, Bhavi Trivedi, MD, Lakshmi Kattamuri, MD. This Post-Counseling Questionnaire is the original creation of the lead three authors of this publication and is not subject to any licensure.

Item	Question	Response Options
1	Has anyone counseled you before regarding your drinking habits?	Yes / No
2	This conversation provided education on alcohol drinking habits.	Strongly disagree / Disagree / Neither agree nor disagree / Agree / Strongly agree
3	It was surprising to you that your alcohol drinking habits are considered unhealthy.	Strongly disagree / Disagree / Neither agree nor disagree / Agree / Strongly agree
4	Discussing your alcohol use habits made you uncomfortable.	Strongly disagree / Disagree / Neither agree nor disagree / Agree / Strongly agree
5	Will you be implementing changes to your drinking habits?	Strongly disagree / Disagree / Neither agree nor disagree / Agree / Strongly agree
6	If answer is neither agree nor disagree, disagree, or strongly disagree, what is the reason? (You do not believe there is an issue; You do not want to cut down on drinking; Monetary cost of quitting; Other)	Open-ended (please specify)
